# epLSAP-Align: a non-sequential protein structural alignment solver with entropy-regularized partial linear sum assignment problem formulation

**DOI:** 10.1093/bioinformatics/btaf309

**Published:** 2025-05-20

**Authors:** Xuechen Zhang, Zhuoyang Chen, Junyu Li, Qiong Luo, Longjun Wu, Weichuan Yu

**Affiliations:** Department of Electronic and Computational Engineering, The Hong Kong University of Science and Technology, Hong Kong SAR, China; Data Science and Analytics Thrust, Information Hub, The Hong Kong University of Science and Technology (Guangzhou), Guangzhou, Guangdong 511400, China; Department of Ocean Science and Center for Ocean Research in Hong Kong and Macau, The Hong Kong University of Science and Technology, Hong Kong SAR, China; Data Science and Analytics Thrust, Information Hub, The Hong Kong University of Science and Technology (Guangzhou), Guangzhou, Guangdong 511400, China; Department of Computer Science and Engineering, The Hong Kong University of Science and Technology, Hong Kong SAR, China; Department of Ocean Science and Center for Ocean Research in Hong Kong and Macau, The Hong Kong University of Science and Technology, Hong Kong SAR, China; Department of Electronic and Computational Engineering, The Hong Kong University of Science and Technology, Hong Kong SAR, China

## Abstract

**Motivation:**

The three-dimensional protein tertiary structure alignment is a fundamental problem that seeks insights into functions and evolution. Previous structure alignment algorithms have adopted the sequential assumption and used dynamic programming solvers. However, many distantly related structures exhibit non-sequential similarities, and non-sequential alignment tools are less efficient and accurate than sequential ones. In this paper, we formulate the non-sequential alignment as the **E**ntropy-regularized **P**artial **L**inear **S**um **A**ssignment **P**roblem (epLSAP) and propose a solver based on Sinkhorn algorithms, referred to as epLSAP-Align.

**Results:**

Compared with existing non-sequential alignment solvers, our epLSAP-Align can explicitly model the gap penalty, efficiently achieve global optimality and balance coverage and fidelity. We show that epLSAP-Align can be easily integrated into the existing frameworks, such as TM-align and MICAN, resulting in the non-sequential alignment tool epLSAP-TM and epLSAP-MICAN, respectively. Both epLSAP-TM and epLSAP-MICAN achieve better performance than the existing non-sequential alignment tools in terms of biologically meaningful structure overlaps on two sequential alignment test sets MALIDUP and MALISAM, and four non-sequential alignment test sets MALIDUP-ns, MALISAM-ns, 64-difficult-case and RIPC datasets. Also, compared with the most recent non-sequential alignment tool USalign2, our epLSAP-TM is at least 22% faster under the same setting.

**Availability and implementation:**

Our source code is available at https://github.com/xzhangem/epLSAP-align.

## 1 Introduction

Comparisons and alignments of three-dimensional protein structures at the atomic level resolution are important in computational biology and bioinformatics because protein structures are usually more conserved than sequences (Illergård *et al.* 2009), and enable diverse applications such as protein classification (Murzin *et al.* 1995), evolutionary relationship analysis (Barrio-Hernandez *et al.* 2023), protein functional prediction (Gligorijević *et al.* 2021), molecular analysis (Kuntz *et al.* 1994) and drug discovery (Borkakoti and Thornton 2023). To a large extent, structural similarity implies functional similarities, and unannotated functions of a protein can be predicted by comparing them with structurally similar proteins with known functions (Hasegawa and Holm 2009, Ma and Wang 2014).

Protein structure alignment has been studied for over forty years. Existing tools can be categorized into sequential (SQ) alignment and non-sequential (NS) alignment (Yuan and Bystroff 2005, Ma and Wang 2014). In sequential alignment, for any two aligned residue pairs *i*–*j* and $i^{\prime }$–$j^{\prime }$ from two protein structures, if $i<i^{\prime }$ then $j<j^{\prime }$ (Strickland *et al.* 2005). Such an ordering relationship enables efficient search in dynamic programming solvers. Existing sequential alignment algorithms take two different approaches to exploring features in protein similarity measurement: flexible local feature alignment and rigid-body superposition optimization. Flexible local feature alignments use local features regardless of the superposition of proteins to be aligned. They are good at comparing proteins in different conformational states and locally identifying conserved regions. Representative flexible methods include 3D-BLAST (Yang and Tung 2006), CLePAPS (Wang and Zheng 2008), Kpax (Ritchie *et al.* 2012) and Foldseek (Van Kempen *et al.* 2024). In comparison, rigid-body superposition-based methods treat structures as rigid bodies and explore similarities by comparing their shapes. They are well suited for closely evolutionarily related protein comparisons. Representative rigid-body superposition-based methods include TM-align family (Zhang and Skolnick 2005, Zhang *et al.* 2022, Margelevičius 2024), SSM (Krissinel and Henrick 2004), CE (Shindyalov and Bourne 1998), DALI (sequential version) (Holm 2022), DeepAlign (Wang *et al.* 2013) and SPalign (Yang *et al.* 2012). Furthermore, many tools (Ritchie *et al.* 2012, Wang *et al.* 2013) adopt both flexible alignment and rigid body-based alignment to produce more comprehensive alignment results than those with the single methodology.

Over 17% of structurally similar proteins whose fragment rearrangement can only be identified by non-sequential alignments (Abyzov and Ilyin 2007). Common non-sequential alignments include circular permutation and local swap, and they are preferred in cases such as binding interface comparisons for evolutionary and functional inference, where the overall similarity of protein pairs may be more informative than sequential similarity. Representative non-sequential alignment tools include GANGSTA+ (Guerler and Knapp 2008), FlexSnap (Salem *et al.* 2010), CLICK (Nguyen and Madhusudhan 2011), MICAN (Minami *et al.* 2013, 2018), SPalignNS (Brown *et al.* 2016), FTAlign (Wen *et al.* 2020) and USalign2 (Zhang and Pyle 2022).

Among non-sequential methods, CLICK and GANGSTA+ focus on helices and strands features and use combinatorial search algorithms. FlexSnap adopts a greedy search strategy on fragment pairs. MICAN and FTAlign are top-performers in topology-independent superposition. SPalignNS and USalign2 break the ordering requirement of dynamic programming and use the asymmetric form of linear sum assignment problem (LSAP) (Burkard and Cela 1999), the general case of sequential alignment, for non-sequential search. To reduce the computational cost of the exact solution, SPalignNS uses the deep greedy switching (DGS) (Naiem and El-Beltagy 2013) and USalign2 uses the enhanced greedy search (EGS) algorithm (Hu *et al.* 2018).

These non-sequential methods, however, suffer from the following three drawbacks associated with the greedy search. First, the above-mentioned greed search methods are heuristic algorithms and global optimality is not guaranteed. Second, as studied in sequential alignment, the gap penalty for deletion/insertion is essential, mainly because it facilitates the matching for various biologically meaningful results. However, neither of the above greedy methods explicitly models of gap penalty, and deletion/insertion is checked during heuristic assignments, which may lead to a suboptimal solution. Third, existing non-sequential methods mainly focus on the fidelity (minimal average spatial deviations among aligned pairs) and disregard the overall coverage (maximal aligned residue numbers). Generally, the best fidelity of the minimum average spatial deviations and the maximum overall coverage cannot be achieved at the same time, and one increases at the cost of the other. An important topic in sequential alignment, the coverage-fidelity trade-off control, which emphasizes producing an alignment with the smallest deviation with as many aligned pairs as possible (Zemla 2003, Krissinel and Henrick 2004, Collier *et al.* 2017), can be further explored in non-sequential alignments. Therefore, we aim to consider the solutions to the above issues in non-sequential alignment.

To address these challenges, we propose epLSAP-Align for non-sequential alignment with three advantages of the sequential dynamic programming search algorithm, including explicit alignment gap modeling, efficient global optimality and balancing coverage-fidelity trade-off control. Specifically, epLSAP-Align uses entropy regularized optimal transport solvers (Sinkhorn 1967, Cuturi 2013, Brun *et al.* 2022) for both efficiently solving partial LSAP (pLSAP) and balancing coverage-fidelity trade-offs, and is integrated into two representative superposition alignment algorithms: topology-independent method MICAN Minami *et al.* (2013) and topology-dependent method TM-align (Zhang and Skolnick 2005). Comprehensive experiments on seven public datasets for non-sequential alignment demonstrate the effectiveness and efficiency of our proposed epLSAP-Align.

## 2 Materials and methods

### 2.1 epLSAP for non-sequential protein structural alignment

#### 2.1.1 Gap modeling in non-sequential alignment

**Figure 1.**
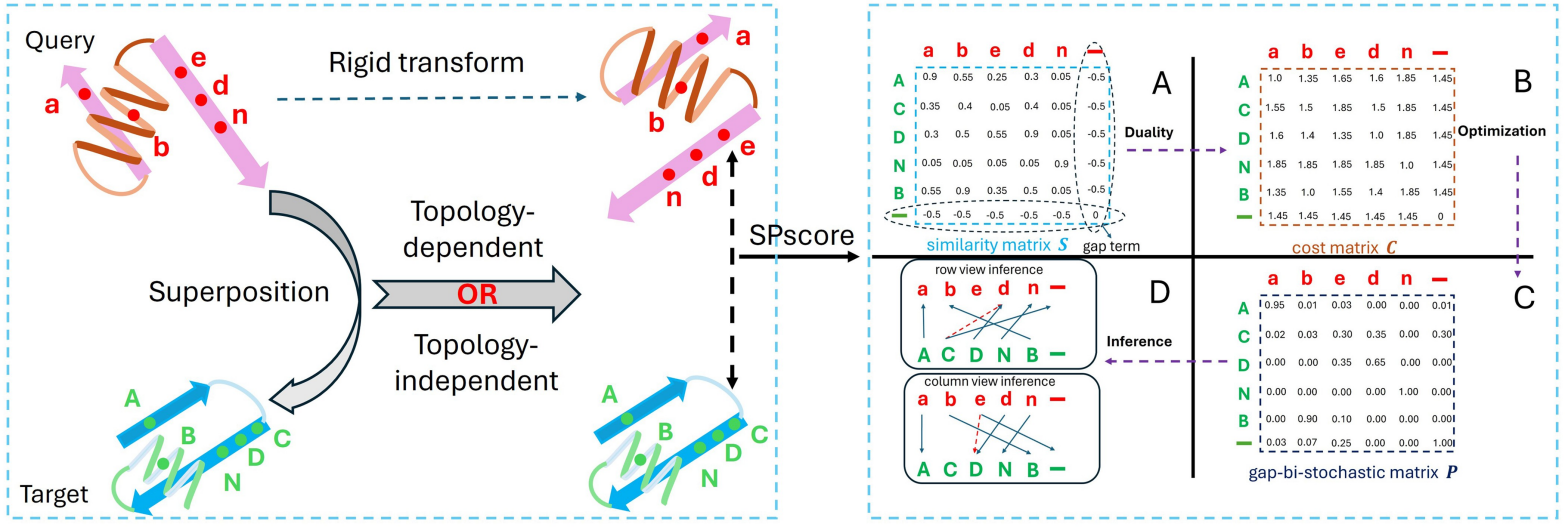
Graphical illustration of the epLSAP-Align workflow using an example pair abedn- ACDNB. Our goal is to align identical letters regardless of their cases. The left panel shows that epLSAP-Align can be integrated into a topology-dependent superposition method (e.g. TM-align) or a topology-independent superposition method (e.g. MICAN) before non-sequential alignment. After superposition, we calculate the SP-score (Yang *et al.* 2012) for similarity measurement. The right panel shows the non-sequential alignment procedure of epLSAP-Align with the given similarity matrix, and the calculation is detailed in Supplementary Section S1.

We illustrate the proposed epLSAP-Align with a simple example in Fig. 1. Here we focus on the right panel about non-sequential solver formulation. Given two sequences $u=\{u_{1},\ldots ,u_{m}\}$ and $v=\{v_{1},\ldots ,v_{n}\}$, to model the gap penalty during sequence matching, we add the element ‘-’ to the end of each sequence representing a gap term, and the revised sequences are $u_{0}=\{u_{1},\ldots ,u_{m},u_{m+1}\}$ and $v_{0}=\{v_{1},\ldots ,v_{n},v_{n+1}\}$ with $u_{m+1}=v_{n+1}=$ “–.” The non-sequential alignment with gap penalty corresponds to some invertible mapping $\varphi :U_{0}\to V_{0}$ with the following property


(1) $$\begin{array}{cc} & \forall 1\leq i\leq m,\;|\varphi (u_{i})|=1,\;\forall 1\leq j\leq n,\;|\varphi^{-1}(v_{j})|=1,\\ & u_{m+1}\in \varphi^{-1}(v_{n+1}),\;v_{n+1}\in \varphi (u_{m+1}), \end{array}$$

where the first line indicates that each element of u and v corresponds to an element in another sequence or “–,” and the second line indicates that a “–” has at least one image or preimage. In the LSAP optimizing diagram, the correspondence map to be solved between u and v is represented by the bi-permutation matrix $M\in {\left\{0,1\right\}}^{m\times n}$, where the one-to-one match between the *i*-th element of u and the *j*-th element of v is represented by $M_{i,j}=1$ and mismatch as $M_{i,j}=0$. In the pLSAP setting, we can modify **M** in LSAP into the gap-bi-permutation matrix $X\in {\left\{0,1\right\}}^{\left(m+1)\times (n+1\right)}$ to represent the correspondence map φ of Equation (1) in the matrix form as follows


(2) $$\begin{array}{cc} & \sum_{j=1}^{n+1}X_{p,j}=\sum_{i=1}^{m+1}X_{i,q}=1,\;X_{p,j},\;X_{i,q}\in \{0,1\},\\ & \forall 1\leq p\leq m,\;1\leq q\leq n,\;\text{and}\;X_{m+1,n+1}=1, \end{array}$$

where $X_{i,j}=1,\;(i,j)\neq (m+1,n+1)$ denotes the correspondence of the *i*-th element of $u_{0}$ and the *j*-th element of $v_{0}$, and $X_{m+1,n+1}=1$ stands for the gap-to-gap correspondence. In structural alignment settings, the similarity matrix $S\in R_{+}^{\left(m+1)\times (n+1\right)}$ consists of the similarity scores between residues and gap penalties. For 1≤i≤m, 1≤j≤n, $S_{i,j}$ is the similarity score between $u_{i}$ and $v_{j}$; $S_{m+1,j}$ and $S_{i,n+1}$ are the gap penalties; $S_{m+1,n+1}$ is set to zero because it makes no difference to the result. The pLSAP for non-sequential protein alignment reads


(3) $$\begin{array}{cc} & \underset{X}{\max}\sum_{i=1}^{m+1}\sum_{j=1}^{n+1}S_{i,j}X_{i,j},\;s.t.\;\sum_{j=1}^{n+1}X_{p,j}=\sum_{i=1}^{m+1}X_{i,q}=1,\\ & \forall \;1\leq p\leq m,\;1\leq q\leq n,\;X_{m+1,n+1}=1, \end{array}$$

where maximizing term indicates the objective of finding the optimal correspondence *w.r.t.* the largest total similarity, and equality constraints come from Equation (2). We then transform Equation (3) into its dual form for minimization. With positive coefficients $\lambda_{i}$ (1≤i≤m), $\sigma_{j}$ (1≤j≤n), and *k*, the term to be minimized reads


$$\begin{array}{cc} & \sum_{i=1}^{m}\lambda_{i}\sum_{j=1}^{n+1}X_{i,j}+\sum_{j=1}^{n}\sigma_{j}\sum_{i=1}^{m+1}X_{i,j}+kX_{m+1,n+1}-\sum_{i=1}^{m+1}\sum_{j=1}^{n+1}S_{i,j}X_{i,j}\\ := & \sum_{i=1}^{m+1}\sum_{j=1}^{n+1}\left(D_{i,j}-S_{i,j}\right)X_{i,j}:=\sum_{i=1}^{m+1}\sum_{j=1}^{n+1}C_{i,j}X_{i,j}, \end{array}$$

where


(4) $$\begin{array}{cc} & D_{i,j}=\{\begin{array}{c} \lambda_{i}+\sigma_{j},1\;\leq \;i\;\leq \;m,1\;\leq \;j\;\leq \;n\\ \lambda_{i},j=n+1,\\ \sigma_{j},i=m+1,\\ k,i=m+1,j=n+1. \end{array} \end{array}$$

The new matrix C is the cost matrix. To ensure $C_{i,j}\;\geq \;0$, we can simply take all $\lambda_{i}$ and $\sigma_{j}$ as $c=\max_{i,j}S_{i,j}+\epsilon$, and k=0.

#### 2.1.2 epLSAP and sinkhorn solver

To reduce the computation of the combinatorial optimization problem Equation (4), we first use the stochastic matrix to approximate the permutation matrix. In our setting, the gap-bi-stochastic matrix $P\in {\left[0,1\right]}^{\left(m+1)\times (n+1\right)}$ is the continuous version of X. With the stochastic matrix surrogate, the dual form Equation (4) is a partial optimal transport (pOT) problem, and it has been well studied that the Sinkhorn algorithm can efficiently offer a decent approximate solution for entropy-regularized pOT problems. Formally, entropy-regularized pOT reads


(5) $$\begin{array}{cc} & \underset{P}{\min}\sum_{i=1}^{m+1}\sum_{j=1}^{n+1}\left(C_{i,j}P_{i,j}+\frac{1}{\lambda }P_{i,j}\;\text{log}\;P_{i,j}\right),\\ s.t. & \sum_{j=1}^{n+1}P_{i,j}=1,\;\forall 1\;\leq \;i\;\leq \;m,\;\sum_{i=1}^{m+1}P_{i,j}=1,\;\forall 1\;\leq \;j\;\leq \;n,\\ & P_{m+1,n+1}=1, \end{array}$$

where $H(P):=\sum_{i,j}^{m+1,n+1}P_{i,j}\;\text{log}\;P_{i,j}$ is the entropy of P, and λ is the parameter to control the influence of the entropy term on the pOT solution. By using the Lagrange multiplier $a_{i}$ and $b_{j}$, the optimal solution $P^{*}$ satisfies


(6) $$\begin{array}{c} P_{i,j}^{*}=\{\begin{array}{cc} e^{-\frac{1}{2}-a_{i}}e^{-\lambda C_{i,j}}e^{-\frac{1}{2}-b_{j}}, & (i,j)\neq (m+1,n+1),\\ 1, & (i,j)=(m+1,n+1). \end{array} \end{array}$$

After getting the optimal gap-bi-stochastic matrix $P^{*}$, we can infer the alignment by taking indices with maximum $P^{*}$ values from the row or column directions and eliminating the non-unique correspondence. The alignments from both row and column directions are meaningful and largely similar in most cases. We can choose results in either row or column direction by costumed criteria. In this study, we choose the alignment with the higher TM-score (Zhang and Skolnick 2004) as default. The Sinkhorn solver for $P^{*}$ and alignments is in Supplementary Algorithm S1, also with a concrete computational example attached. Previous analysis (Cuturi 2013) shows that the time complexity of Sinkhorn algorithm is $O(n^{2})$, where *n* is the maximum length of the two proteins. Compared with previous LSAP heuristic approximation algorithms for non-sequential alignment, whose theoretical time complexity is also $O(n^{2})$, our epLSAP benefits from its insensitivity to initialization. Specifically, greedy search algorithms such as those used in USalign2 depend on initial seeds, and selecting different initial seeds will lead to different results. To eliminate the effect of initial seeds, more than one round of greedy search is performed. In contrast, the convergence of epLSAP regardless of initialization is ensured by the property of the Sinkhorn algorithm, whereas USalign2 uses greedy search with different initializations and chooses the best.

#### 2.1.3 Entropy regularization and coverage-fidelity trade-off

The entropy regularization in Equation (5) not only provides an efficient solver for pLSAP, but is also a biologically meaningful controller for coverage-fidelity trade-offs in protein structural alignment. In the assessment of structural alignment, the number of aligned residues (Nali), and the spatial or geometrical similarity across aligned residues, such as root mean square deviation (RMSD), namely coverage and fidelity metrics, respectively, usually yield inconsistent results during optimization. For example, aligning all residues will lead to the best Nali but poor RMSD because all the implausible alignments are included. In contrast, only picking the nearest aligned pairs will lead to the best RMSD but the worst Nali. Because coverage-fidelity trade-offs vary in different applications, it is desirable that the function to be optimized can be tunable for coverage-fidelity trade-offs. In Equation (5), the pOT part corresponds to maximizing fidelity by aligning the pairs with the smallest costs, and the entropy part aims to control coverage by maximizing the entropy H(P). When H(P) is small, it indicates that the majority mass of P is concentrated on a small portion of entries in P, and the alignment inferred from such a low entropy P will lead to a small Nali. In contrast, a high-entropy P uniformly distributes the mass to each entry and increases the probability of finding the aligned pair of each residue, leading to a large Nali. Therefore, the coverage-fidelity trade-off for alignment can be tunable by setting λ. When λ is among plausible intervals for the Sinkhorn solver, a smaller λ indicates more weight on coverage, and a larger λ indicates more focus on fidelity. Our experimental results in Section 3.5 confirm these analysis.

### 2.2 Implementation of epLSAP-Align

To demonstrate the flexibility of the epLSAP-Align as an accurate and efficient solver for non-sequential protein structural alignment with different superposition methods, we integrate it into two representative structural alignment frameworks: MICAN (Minami *et al.* 2013) and TM-align (Zhang and Skolnick 2005). MICAN is the representative method for using the greedy search strategy on the secondary structural element (SSE) for topology-independent superposition. TM-align is the representative method for using Kabsch method-based topology-dependent superposition. We implement epLSAP-Align by first using the superposition estimated from MICAN and TM-align, denoted as epLSAP-MICAN and epLSAP-TM, respectively. Then with the superposed structures, we adopt SP-score (Yang *et al.* 2012) to calculate the score matrix for alignment optimization as follows


(7) $$\begin{array}{c} \text{SP-score}:=\frac{1}{L^{1-\alpha }}\sum_{d_{ij}<2d_{0}}^{n}\left(\frac{1}{1+d_{ij}^{2}/d_{0}^{2}}-0.2\right), \end{array}$$

where *L* is the sum of aligned core residues, scale factor α=0.3, and size-independent normalization factor $d_{0}=4Å$. The SP-score is used for the final alignment because its fixed normalization factor $d_{0}$ ensures that only sufficiently close residue pairs are considered as candidates. This leads to more robust results compared to size-dependent normalization scores [e.g. TM-score (Zhang and Skolnick 2004)], particularly when exploring non-sequential structural correspondence between large proteins. The default parameters in Supplementary Algorithm S1 are λ=100, ϵ=0.5 and $T_{\max}=5000$.

### 2.3 Evaluation metrics for non-sequential alignment

To assess the non-sequential alignment quality, we first use three standard reference-independent metrics (Brown *et al.* 2016, Wen *et al.* 2020): the number of aligned residues (Nali), root mean square deviation (RMSD), and structure overlap (SO). Given query protein *Q* and target protein *T* with *n* aligned residues $\left\{q_{1},\ldots ,q_{n}\right\}$ and $\left\{t_{1},\ldots ,t_{n}\right\}$, respectively, where n ≤ min{|Q|,|T|}, the Euclidean distance between the *i*-th aligned residue after superposition is $d_{i}$, the formal definitions of Nali, RMSD and SO between *Q* and *T* read


(8) $$\begin{array}{cc} & \text{Nali}:=n,\\ & \text{RMSD}:=\sqrt{\frac{1}{n}\sum_{i}^{n}d_{i}^{2}},\;\text{and}\\ & \text{SO}:=100\times \frac{1}{n}\sum_{i=1}^{n}1(d_{i}\;\leq \;3.5), \end{array}$$

where $1(d_{i}\;\leq \;3.5)$ is the indicator function on judging whether $d_{i}$ is less than 3.5Å. Here, Nali measures the coverage, RMSD measures the fidelity, and SO measures the performance on the coverage-fidelity trade-off. To further explore how the aligned residues distribute across different RMSD thresholds, we expand the classical SO into a function about structure overlap *w.r.t.* varied RMSD threshold as follows


(9) $$\begin{array}{c} \text{SO}\_\text{th}(t):=100\times \frac{1}{n}\sum_{i=1}^{n}1(d_{i}\;\leq \;t). \end{array}$$

Following previous work (Brown *et al.* 2016, Zhang and Pyle 2022), we also adopt two reference-dependent metrics. The first is equivalent reference residues (EQR), defined as the total number of aligned residue pairs shared by the manually curated reference alignment and the alignments from automated alignment tools. The second is the percentage of agreement, defined as EQR divided by the length of the reference alignment.

### 2.4 State-of-the-art tools for comparisons

To evaluate the performance of the proposed epLSAP-Align, we use six state-of-the-art structure alignment tools for comparison. Five of these six tools are the most recent non-sequential structure alignment tools, including USalign2 (Zhang *et al.* 2022), FTAlign (Wen *et al.* 2020), SPalignNS (Brown *et al.* 2016), MICAN (Minami *et al.* 2013) and CLICK (Nguyen and Madhusudhan 2011). In USalign2, there are two non-sequential aligning modes. USalign2 (fNS) treats residues as point clouds and abandons any sequential order information, and USalign2 (sNS) preserves the sequential order within aligned fragment pairs. Also, we choose two widely used tools, TM-align (Zhang and Skolnick 2005) and SSM (Krissinel and Henrick 2004), which has decent performance even in both sequential and non-sequential cases. To use these methods for comparisons, we download their latest versions and run them locally with the default parameters.

### 2.5 Non-sequential alignment test datasets

To evaluate the non-sequential alignment performance of our epLSAP-Align, we use seven widely used datasets, including MALIDUP, MALISAM, MALIDUP-NS, MALISAM-NS, RIPC, 64-difficult-case, and HOMSTRAD. We summarize each dataset in Supplementary Section S2.

## 3 Results

### 3.1 Reference-independent evaluations

Table 1 shows the reference-independent comparisons of the representative non-sequential alignment methods. We also include the widely used sequential alignment method TM-align as a reference to compare the performance improvement from non-sequential methods applied on datasets with great extents of NS. Except for the HOMSTRAD dataset, either epLSAP-TM or epLSAP-MICAN achieves the best SO scores, which balance Nali and RMSD. On the HOMSTRAD dataset, our epLSAP-TM and epLSAP-MICAN perform slightly worse than SPalignNS on SO, but better than the other methods. Noticeably, our epLSAP-MICAN is based on the superposition matrix of MICAN but with the proposed epLSAP-Align, and epLSAP-MICAN performs better than the original MICAN. Similarly, USalign2 uses the greedy algorithm for heuristic search whereas epLSAP-TM uses epLSAP-Align for global optimization instead. The improved SO performance of epLSAP-MICAN and epLSAP-TM indicates that the proposed epLSAP algorithm can be combined with various existing (non-)sequential structural alignment frameworks, and the explicit controlling of coverage-fidelity trade-off of epLSAP helps produce results with better structural overlap.

**Table 1.** Comparisons *w.r.t.* Reference-independent metrics Nali, RMSD (Å) and SO (%) on MALIDUP(-ns), MALISAM(-ns), HOMSTRAD, 64-difficult-case and RIPC dataset, respectively.

Dataset	Metric	Method									
		epLSAP-TM	epLSAP-MICAN	USalign2 (fNS)	USalign2 (sNS)	FTAlign	MICAN	SPalignNS	CLICK	TM-align	SSM
MALIDUP	Nali	89	88	93	92	87	85	63	61	87	80
	RMSD	2.42	2.40	2.75	2.71	2.68	2.58	1.61	1.81	2.69	2.30
	SO(%)	**75.8** ✔	**76.2** ✔	74.5	74.9	73.0	67.2	62.3	62.3	70.4	66.2
MALISAM	Nali	65	65	69	69	64	62	35	35	63	57
	RMSD	2.72	2.66	3.19	3.18	3.12	2.99	1.82	1.95	3.15	2.75
	SO(%)	**70.8** ✔	**71.0** ✔	69.1	69.3	67.0	50.5	46.4	44.0	63.0	58.6
MALIDUP-ns	Nali	86	86	92	93	86	84	58	60	62	54
	RMSD	2.63	2.53	2.87	2.83	2.73	2.55	1.69	1.81	3.01	2.31
	SO(%)	70.7	**72.3** ✔	70.1	70.4	**71.4**	66.6	57.7	58.6	47.5	47.1
MALISAM-ns	Nali	61	60	67	64	62	60	31	34	48	42
	RMSD	2.55	2.64	3.28	3.24	3.13	2.96	1.82	1.94	3.44	2.72
	SO(%)	**66.8** ✔	**65.4** ✔	65.1	64.8	65.0	63.2	40.9	42.9	45.7	44.5
HOMSTRAD	Nali	168	167	172	171	168	167	155	153	169	159
	RMSD	1.96	1.97	2.15	2.14	2.07	2.01	1.41	1.50	2.05	1.69
	SO(%)	**88.0** ✔	87.9 **✔**	86.7	86.6	86.5	83.8	**88.1**	86.3	85.4	84.5
64-difficult-case	Nali	79	78	83	81	83	80	57	51	82	70
	RMSD	2.67	2.70	3.03	3.09	2.98	2.90	1.78	1.94	2.97	2.59
	SO(%)	**69.7**	**69.5** ✔	69.3	69.1	69.1	59.8	56.9	48.5	66.5	62.2
RIPC	Nali	157	155	179	168	155	146	130	123	135	112
	RMSD	2.58	2.58	3.30	3.57	3.28	3.06	1.91	1.97	3.47	2.63
	SO(%)	**65.5**	63.8 ✔	**65.2**	58.0	55.4	56.5	65.1	63.1	48.1	46.1

Note: The best SO performance is **bold and underlined**, and the second best is **bold**. We take USalign2 (fNS) and MICAN as the comparison baseline for epLSAP-TM and epLSAP-MICAN, respectively. SO metrics followed with ✔ indicate statistically significant improvements to baselines (two-sample *t*-test, p<0.05).

We use the two-sample t-test and binary plot (Brown *et al.* 2016) to show the significance of the SO improvement brought by the epLSAP solver. The baseline of epLSAP-TM is Usalign2 (fNS) and the baseline of epLSAP-MICAN is MICAN, respectively. Table 1 shows that except those of epLSAP-TM on MALIDUP-ns, 64-difficult-case and RIPC datasets, all comparison results are statistically significant with p<0.05. Because epLSAP-MICAN performs significantly better than MICAN, we therefore focus on the detailed distribution comparison *w.r.t.* the SO improvement between epLSAP-TM and USalign2, as shown in Supplementary Fig. S3. For 68.1%∼86.7% samples of the datasets used, epLSAP-TM performs better than USalign2 (fNS).

To better show how the aligned residues distribute across different RMSD thresholds, we use the varying structure overlaps *w.r.t.* RMSD in Equation (9). Here we vary the threshold from 3.0 to 7.0 and record the corresponding overlap at intervals of 0.5 in Supplementary Fig. S4. We can see that SO_th curves of both CLICK and SPalignNS are much more stable than others. Also taking their properties of lower Nali and RMSD, as shown in Table 1, we can conclude that CLICK and SPalignNS are specialized in good fidelity results, where the aligned residues are very close after superposition. TM-align is the sequential method and its SO_th performs not well in non-sequential datasets. Among the remaining methods, our epLSAP-TM and epLSAP-MICAN achieve higher SO_th with small thresholds, and we show this result in Supplementary Fig. S4 by specifying two vertical lines when the threshold is 3.5Å and 5.0Å, the latter 5.0Å is another important threshold to quantify the structure overlap (Zhang and Skolnick 2005). As RMSD threshold increases over 5.0Å, methods with higher Nali than our epLSAP-TM (MICAN), including USalign2 (fNS) and USalign2 (sNS), achieve higher SO_th than epLSAP-TM (MICAN). FTAlign shares a similar Nali with epLSAP-TM (MICAN), and at a high RMSD threshold level (>6.5Å), it begins to achieve a similar or higher SO_th than epLSAP-TM (MICAN). epLSAP-MICAN improves MICAN on both Nali and RMSD metrics. All these results contribute to the conclusion that the proposed epLSAP solver can help obtain the alignment results with a better coverage-fidelity trade-off.

### 3.2 Reference-dependent evaluations

We conduct reference-dependent evaluations on three datasets with non-sequential annotation: RIPC, MALIDUP-ns and MALISAM-ns. RIPC annotation is relatively sparse with only 282 annotated pairs. MALIDUP-ns and MALISAM-ns, two artificial non-sequential datasets, have 18837 and 7370 annotated pairs, respectively. When evaluated with the RIPC dataset, as shown in Table 2, our method epLSAP-MICAN and CLICK produce the second highest EQR, and epLSAP-TM performs slightly better than its most similar competitor USalign2 (fNS). For more detailed comparisons, we include the EQR of each sample pair in the RIPC dataset in Supplementary Table S2, in which we can see that for pair **d1nkl___d1qdma1**, USalign2 (fNS and sNS), SPalignNS and CLICK performs much better than others, and aligned pairs of **d1nkl d1qdma1** take a large portion (72 out of 282), which makes USalign2 (sNS) perform much better than our epLSAP-based methods. For the remaining samples, our epLSAP-based methods have slightly better overall performance than the others. Experiments on MALIDUP-ns and MALISAM-ns datasets are at their much larger scales, as shown in the second and third rows of Table 2. In MALIDUP-ns and MALISAM-ns, our epLSAP-MICAN achieves the best EQR, and epLSAP-TM is better than USalign2 (fNS).

**Table 2.** Reference-dependent comparisons *w.r.t.* EQR/Agr(%) on the annotated non-sequential datasets.

Method	epLSAP-TM	epLSAP-MICAN	USalign2 (fNS)	USalign2 (sNS)	FTAlign	MICAN	SPalignNS	CLICK	TM-align	SSM
RIPC	192/68.1	194/68.8	191/67.7	229/81.2	185/65.6	184/65.2	192/68.1	194/68.8	132/46.8	149/52.8
MALIDUP-ns	12223/64.9	12542/66.6	12194/64.7	12319/65.4	9532/50.6	9826/52.2	8950/47.5	9062/48.1	6231/33.1	7807/41.4
MALISAM-ns	2413/32.7	2797/38.0	2348/31.9	2565/34.8	2189/29.7	2516/34.1	1986/26.7	2032/27.6	1442/19.6	1676/22.7

### 3.3 Computational efficiency

To fairly evaluate the computational efficiency of the proposed epLSAP-Align, we compare the average CPU time of epLSAP-TM with the other methods, especially focusing on USalign2 in the same programming environment because it is one of the fastest non-sequential alignment methods. The CPU environment for all experiments is Intel(R) Xeon(R) Gold 6130 CPU @ 2.10 GHz core, and the CPU time is summarized in Table 3. From the results, we can see that on average our epLSAP-TM is 22.5% faster than USalign2 (sNS), and 44.9% faster than USalign2 (fNS). We also include the average CPU time of TM-align and SSM, two widely used alignment methods not restricted in non-sequential cases. Although non-sequential methods achieve better performance *w.r.t.* the above non-sequential evaluation metrics, we observe that non-sequential methods are much slower than TM-align and SSM, and SSM is significantly faster than others.

**Table 3.** Average CPU time (sec) comparisons between epLSAP-TM and USalign2 (sNS and fNS) on seven datasets.

Method	MALIDUP	MALISAM	MALIDUP-ns	MALISAM-ns	HOMSTRAD	64-diffcult	RIPC	Total average
epLSAP-TM	0.058	0.057	0.058	0.056	0.334	0.076	0.254	0.313
USalign (sNS)	0.066	0.065	0.067	0.066	0.432	0.087	0.378	0.404
USalign (fNS)	0.105	0.102	0.107	0.101	0.658	0.169	0.731	0.615
FTAlign	2.838	2.515	2.822	2.546	8.174	3.231	4.821	7.747
MICAN	0.157	0.102	0.156	0.103	1.175	0.156	0.316	1.093
SPalignNS	0.618	0.426	0.622	0.441	2.954	0.732	1.523	2.766
CLICK	5.527	2.214	5.651	2.229	8.654	8.476	5.753	8.348
TM-align	0.016	0.017	0.016	0.016	0.079	0.022	0.065	0.074
SSM	0.005	0.005	0.005	0.005	0.009	0.006	0.007	0.008

### 3.4 Discussions on the gap modeling

Structural alignments are partial matching in nature. Give two sequences $u=\{u_{1},\ldots ,u_{m}\}$ and $v=\{v_{1},\ldots ,v_{n}\}$ with m ≤ n, only their subsets $\{u_{\sigma (1)},\ldots ,u_{\sigma (l)}\}\subset u$ and $\{v_{\gamma (1)},\ldots ,u_{\gamma (l)}\}\subset v$ (l ≤ m) are aligned, where σ and γ indicate permutation functions. Classical LSAP formulation without gap modeling aims to obtain the bi-permutation matrix $M\in {\left\{0,1\right\}}^{m\times n}$ indicating whether the *i*-th element of u and *j*-th element of v is matched. Ideally, this formulation will give an *m*-length alignment, where the shorter sequence will be aligned with a subset of the longer one. The deletion and insertion in classical LSAP formulation happen during the inference stage, such as Line 22–27 in Supplementary Algorithm S1 or choosing the pairs with the highest similarity for exchange at each iteration in the greedy algorithm of USalign2 (Hu *et al.* 2018, Zhang and Pyle 2022). In short, we can see that classical LSAP formulation does not fully consider the partial matching nature, and may lead to sub-optimal results. In contrast, the partial LSAP formulation in Equations (1) and (2) models the partial matching by introducing gap terms where all the unaligned elements are matched to the gap terms. Our formulation yields better performance when compared with non-sequential methods in entropy-regularized LSAP (classical LSAP with no gap modeling but with entropy regularizer, denoted as eLSAP-TM and eLSAP-MICAN, respectively), as shown in the Supplementary Table S1.

### 3.5 Coverage-fidelity trade-off by different λ

As discussed in Section 2.1.3, parameter λ can help control coverage-fidelity trade-offs in aligned pairs. Here we use different λ from values 10 to 300 to execute epLSAP-TM and epLSAP-MICAN on the RIPC dataset, and record the corresponding changes of Nali, RMSD, and SO *w.r.t* λ. As shown in Supplementary Fig. S5a and b, Nali and RMSD increase with λ, which indicates that a larger λ [more focus on the entropy term of Equation (5)] indeed leads to alignments of higher coverages (more Nali) but worse fidelity (larger RMSD). Supplementary Figure S5c shows the coverage-fidelity trade-off with an increasing λ. Supplementary Figure S5d shows that SO rapidly becomes stable regardless of the increase of λ, though different coverage-fidelity are shown in Supplementary Fig. S5c. These results demonstrate that our epLSAP-Align shows robust performance *w.r.t.* SO metric with varied λ.

### 3.6 Case study

To further illustrate the advantages of the proposed epLSAP-Align for non-sequential alignment, we use the protein pair **d1af2a1.1–d1af2a1.2** from the manually permuted MALIDUP-ns dataset as an example in Supplementary Fig. S6. In C-H black symbols in line segments are ground truth correspondence, green symbols indicate the algorithm correspondence, and the sizes of these symbols are linearly scaled to the distance between the aligned $C_{\alpha }$ atoms. In this pair, the tail residues of **d1af2a1.1** are manually adjusted to correspond to the head of **d1af2a1.2**, as shown in Supplementary Fig. S6c. In D-F, although our epLSAP-TM starts with the same initial superposition as USalign2 (fNS) and USalign2 (sNS), epLSAP-TM achieves fewer mismatches than both USalign2 (fNS) and USalign2 (sNS). The number of outliers in F is much smaller than those in D-E, and the scales of mismatched green symbols in F are also smaller than those in D-E, indicating that epLSAP-TM produces fewer mismatches with large deviations. As shown in Supplementary Fig. S6g and h, based on the same superposition B, MICAN produces sequential-like alignment, whereas our epLSAP-MICAN produces non-sequential alignment as expected.

We further use the case of the pair **2ES9-1SXJ** in SPalignNS (Brown *et al.* 2016) to illustrate the usefulness of epLSAP solver. We show Nali-RMSD-SO metrics obtained using different methods in the Supplementary Table S3. Our epLSAP-MICAN achieves the best SO performance, and SPalignNS achieves the second best SO performance, with less Nali but significantly lower RMSD. Because epLSAP-MICAN and SPalignNS have different superpositions, to illustrate that the performance gain is brought by the proposed epLSAP solver instead of superposition difference, we further use the proposed epLSAP solver based on the superposition estimated from SPalignNS, and record the results as epLSAP-SP_rigid. When sharing the same superposition as SPalignNS, we can see that our epLSAP-SP_rigid gets the non-sequential alignment with much more Nali at the cost of moderate RMSD increase, thus leading to a better coverage-fidelity trade-off *w.r.t.* the SO metric.

## 4 Conclusion

In this study, we present epLASP-Align, a new non-sequential alignment solver by formulating the alignment problem as the partial linear sum assignment problem and using the Sinkhorn algorithm to solve the entropy-relaxed surrogate. The proposed epLSAP-Align is flexible to be deployed in the existing protein structural alignment frameworks to explore non-sequential correspondences. Compared with the existing non-sequential alignment solvers, epLSAP-Align has three advantages: explicit gap penalty modeling, efficient global optimality and coverage-fidelity trade-offs. To demonstrate the effectiveness, we integrate epLSAP-Align into the existing popular alignment tools such as TM-align and MICAN to form new non-sequential alignment tools, namely epLSAP-TM and epLSAP-MICAN. Both epLSAP-TM and epLSAP-MICAN achieve generally higher biologically meaningful structure overlaps and higher computational efficiency on seven test datasets. Therefore, we expect that epLSAP-Align can be deployed in more alignment frameworks to facilitate the exploration of biologically meaningful non-sequential relationships.

Some further improvements are anticipated. First, suitable post-processing techniques can be developed to refine the resulting alignment. For example, outlier alignments shown in Supplementary Fig. S6f and h can be eliminated by considering pair distance or secondary structure consistency. Secondly, more sophisticated scoring schemes taking secondary structures or internal coordinates into account (Wang *et al.* 2013) may improve the reliability of the results. We also notice that the acceleration of the Sinkhorn-type algorithm for the optimal transport problem is a valuable and popular topic in machine learning and optimization, and recent acceleration algorithms such as (Solomon *et al.* 2015, Alaya *et al.* 2019) leverage the properties of the cost matrix for specific tasks. In the future, an interesting direction is to explore more efficient Sinkhorn-type algorithms with specific considerations of the properties of non-sequential protein structural alignment. A driving forces for developing new methods in structural alignment is their feasibility on the genomic-scale search. Notice that non-sequential methods are still much slower than methods well suited for screening large datasets, such as SSM. Innovative methods enabling non-sequential searching on large-scale datasets are a great direction for future work.

## Supplementary Material

btaf309_Supplementary_Data
