# Supplementary material for: epLSAP-Align: a non-sequential protein structural alignment solver with entropy-regularized partial linear sum assignment problem formulation
Source: Bioinformatics. 2025 May 20;41(6):btaf309. doi: 10.1093/bioinformatics/btaf309 (PMC12137893; doi:10.1093/bioinformatics/btaf309)
Supplement: btaf309_Supplementary_Data [file btaf309_supplementary_data.pdf]

Supplementary Materials

S1 Sinkhorn solver for epLSAP-Align and the computational example

S1.1 Sinkhorn solver

Algo.S1 shows the details of using the Sinkhorn algorithm to solve the optimal gap-bi-stochastic matrix $\mathbf{P}^*$ of Eq. (6) and alignment inference from $\mathbf{P}^*$. In Algo.S1, Line 1∼18 are about solving $\mathbf{P}^*$ using Sinkhorn algorithms, among which Line 4 and 10 are additionally designed to ensure $P_{m+1,n+1} = 1$ at each iteration compared with vanilla entropy-regularized optimal transport [1].

S1.2 A concrete computational example

Here we give a concrete computational example to demonstrate how the epLSAP-Align works for non-sequential alignment. We use the sample of aligning short structures with sequence representation abedn-ACDNB, and our goal is to align identical letters regardless of their cases. Suppose we have determined the superposition by either TM-align [2] or MICAN [3], and calculated the similarity scores. Then the epLSAP-Align will be implemented as follows:

1. Suppose the gap value is -0.5, we extend the original similarity matrix with the gap term into $\mathbf{S}$ as shown in Fig.S1 A;
2. Use the Eq. (4) to transform the similarity matrix $\mathbf{S}$ into the cost matrix $\mathbf{C}$. As shown in Fig.S1 B, we implement this calculation as follows:
    - Take $\max \mathbf{S} = 0.9$ and $\epsilon = 0.05$, and then $\lambda_i = \sigma_j = \max \mathbf{S} + \epsilon = 0.95$ in Eq. (4);
    - For $1 \leq i, j \leq 5$, $C_{i,j} = \lambda_i + \sigma_j - S_{i,j} = 1.9 - S_{i,j}$; for $i = 6$ and $j = 6$ but $(i, j) \neq (6, 6)$, $C_{i,j} = 0.95 - (-0.5) = 1.45$; $C_{6,6} = 0$.
3. With the cost matrix $\mathbf{C}$, we use Line 1∼18 of Algo.S1 to estimate the optimal gap-bi-stochastic matrix $\mathbf{P}^*$, and show results in Fig.S1 C;
4. We infer the alignment from the gap-bi-stochastic matrix from the row view and the column view, respectively (line 19∼29 in Algo.S1), with results shown in Fig.S1 D. Because line-view inference and column-view follows the same criterion only with a different view direction, here we focus on the row-view inference as follows:
    - In row view alignment, from the 1-st to the 5-th row, we take the column element with the maximum $\mathbf{P}$ value, as shown in Line 19∼21 of Algo.S1. After this step, we get the correspondence: A → a; C → d; D → d; N → n; B → b,

and its mapping function in Algo.S1, namely Align_row takes values as follows: Align_row(1) = 1, Align_row(2) = 4, Align_row(3) = 4, Align_row(4) = 5, Align_row(5) = 2.

- For element from the 1-st to the 5-th column, calculate IndxSet(j), $j = 1, ..., 5$ as in Line 23 of Algo.S1, and in our case IndxSet(4) = $\{2, 3\}$ and rest all have one element which indicates one-to-one correspondence.
- For two elements 2 and 3 (correspond to C and D, respectively), as Line 24 and 25 of Algo.S1 defined, we retain the correspondence D $\rightarrow$ d because the probability of D $\rightarrow$ d is $P_{3,4} = 0.65$, which is larger than the probability of C $\rightarrow$ d $P_{2,4} = 0.35$.

In this case, the row-view and column-view inference produce the same alignment results. In practice, the alignments from both row and column directions are meaningful and largely similar, but in some cases, they differ considerably. We can choose results in either row or column direction by customized criteria. In this study, we choose the alignment with the higher TMscore as default.

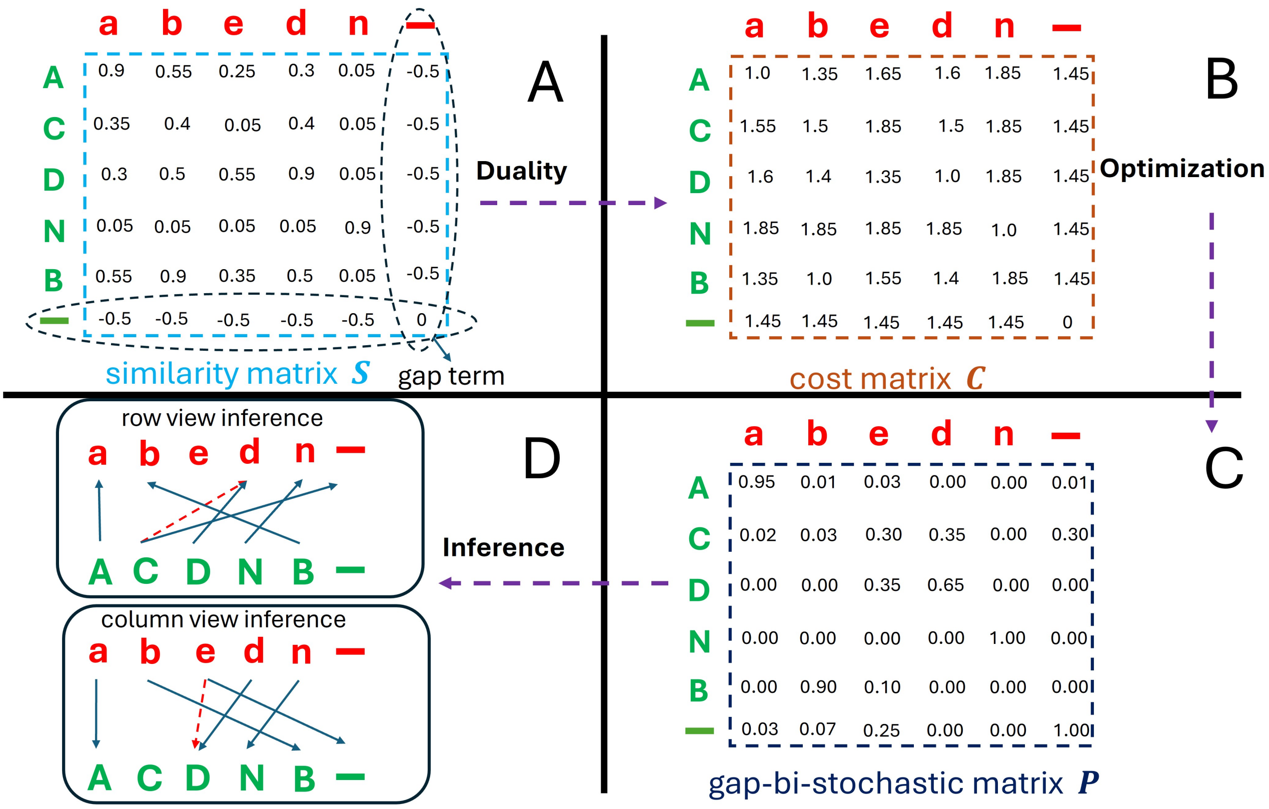

Figure S1: An illustration of the proposed epLSAP-Align with the given similarity matrix of the sequence abedn-ACDNB. Our goal is to align identical letters regardless of their cases. Fig. S1 A shows that we extend the original similarity matrix with the gap term. Fig. S1 B shows that we get the cost matrix from the similarity matrix by computing duality with Eq. (4). Fig. S1 C shows that we get the gap-bi-stochastic matrix to indicate the matching probability from the cost matrix with the Sinkhorn algorithm (Line 1∼18 in Algo.S1). Fig. S1 D shows that we infer the alignment from the stochastic matrix from the row view and the column view, respectively (line 19∼29 in Algo.S1), where the solid lines represent the final match of letters, and the dashed lines represent the non-unique correspondence to be eliminated (Line 22∼27 in Algo.S1).

Algorithm S1 Sinkhorn solver and alignments

Require: Element-wise negative exp of cost matrix $\boldsymbol{K} := e^{-\lambda \boldsymbol{C}}$, tolerant threshold ϵ and maximum iteration $T_{\max}$.

Ensure: Gap-bi-stochastic matrix $\boldsymbol{P}^*$, row-view alignment Align_row and column-view alignment Align_col.

1: Initialize $\boldsymbol{y} \leftarrow \mathbf{1}^{n+1}$, *conv* $\leftarrow$ False, $t \leftarrow 0$.
2: **while** *conv* == False and $t < T_{\max}$ **do**
3:     $\boldsymbol{xp} \leftarrow 1./(\boldsymbol{K}\boldsymbol{y})$; ▷ ./ means element-wise division.
4:     $xp_n \leftarrow 1$; ▷ To meet equality constraint on $P_{m+1,n+1}$ in Eq. (5)
5:     **if** $i \geq 1$ **then**
6:         err_x $\leftarrow ||\boldsymbol{xp}./\boldsymbol{x} - \mathbf{1}^{n+1}||$; ▷ $||\cdot||$: ℓ_2-norm
7:     **end if**
8:     $\boldsymbol{x} \leftarrow \boldsymbol{xp}$;
9:     $\boldsymbol{yp} \leftarrow 1./(\boldsymbol{K}^T\boldsymbol{x})$;
10:     $yp_m \leftarrow 1$; ▷ To meet equality constraint on $P_{m+1,n+1}$ in Eq. (5)
11:     err_y $\leftarrow ||\boldsymbol{yp}/\boldsymbol{y} - \mathbf{1}^{m+1}||$;
12:     $\boldsymbol{y} \leftarrow \boldsymbol{yp}$;
13:     **if** $i \geq 1$ and $\min(\text{err\_x}, \text{err\_y}) < \epsilon$ **then**
14:         *conv* $\leftarrow$ True
15:     **end if**
16:     $i \leftarrow i + 1$;
17: **end while**
18: Update $\boldsymbol{P}^* \leftarrow \text{diag}(\boldsymbol{x})\boldsymbol{K}\text{diag}(\boldsymbol{y})$. ▷ diag($\cdot$): diagonalization.
19: **for** i from 1 to m **do** ▷ Row-view alignment.
20:     Align_row(i) = $\arg\max_{1 \leq j \leq n} P^*_{ij}$.
21: **end for**
22: **for** j from 1 to n **do** ▷ Eliminate non-uniqueness.
23:     IndxSet(j) = $\{i \mid \text{Align\_row}(i) == j\}$.
24:     **if** $|\text{IndxSet}(j)| > 1$ **then**
25:         Retain Align_row(i) with upper-most maximum P^*_{ij}, and map the remaining $i \in \text{IndxSet}(j)$ to the gap term.
26:     **end if**
27: **end for**
28: Repeat Line 22-27 in column view to get Align_col.
29: Return $\boldsymbol{P}^*$, Align_row and Align_col.

S2 Summary of non-sequential alignment test datasets

- **MALIDUP** [4] has 241 pairwise structural alignments for remotely related homologous domains with non-trivial homology. It can be downloaded at http://prodata.swmed.edu/malidup/;
- **MALISAM** [5] has 130 pairwise structural analogous alignments but with distinct SCOP folds. It can be downloaded at http://prodata.swmed.edu/malisam/;
- **MALIDUP-ns** [3] is the artificial non-sequential test sets constructed by applying multiple segment permutation techniques to MALIDUP. It can be downloaded at https://github.com/ShintaroMinami/mican/tree/master/tests/dataset/MALIDUP-ns;
- **MALISAM-ns** [3] is the artificial non-sequential test sets constructed by applying multiple segment permutation techniques to MALISAM. It can be downloaded at https://github.com/ShintaroMinami/mican/tree/master/tests/dataset/MALISAM-ns;
- **HOMSTRAD** [6] contains 398 multi-protein structure alignments, resulting in 9538 pairwise alignments. It can be downloaded at https://mspc.bii.a-star.edu.sg/minhn/results.html;
- **64-difficult-case** [6] contains 64 pairwise alignments from HOMSTRAD with 30% $<$ SO $<$ 70% and RMSD $>$ 2.5. It can be downloaded at https://mspc.bii.a-star.edu.sg/minhn/results.html;
- **RIPC** [7] contains 40 structural pairs with large proportions of **r**epetitions, extensive **i**nsertions/deletions, circular **p**ermutations, and/or considerable **c**onformational variations. We follow the routine of using 20 samples from the RIPC dataset with sequence identities below 0.3. The RIPC references are manually curated, and expert curations focus on non-sequential pairs. It can be downloaded at https://figshare.com/articles/dataset/Benchmark_dataset_of_US-align_NS/20102945/2.

References

[1] M. Cuturi. Sinkhorn distances: Lightspeed computation of optimal transport. *Advances in Neural Information Processing Systems*, 26, 2013.

[2] Y. Zhang and J. Skolnick. Tm-align: a protein structure alignment algorithm based on the tm-score. *Nucleic Acids Res*, 33(7):2302–2309, 2005.

[3] S. Minami, K. Sawada, and G. Chikenji. Mican: a protein structure alignment algorithm that can handle multiple-chains, inverse alignments, c α only models, alternative alignments, and non-sequential alignments. *BMC Bioinformatics*, 14:1–22, 2013.

[4] H. Cheng, B. H. Kim, and N. V. Grishin. Malidup: a database of manually constructed structure alignments for duplicated domain pairs. *Proteins*, 70(4):1162–1166, 2008.

[5] H. Cheng, B. H. Kim, and N. V. Grishin. Malisam: a database of structurally analogous motifs in proteins. *Nucleic Acids Res*, 36(Database issue):D211–D217, 2007.

[6] L. A. Stebbings and K. Mizuguchi. Homstrad: recent developments of the homologous protein structure alignment database. *Nucleic Acids Res*, 32(Database issue):D203–D207, 2004.

[7] G. Mayr, F. S. Domingues, and P. Lackner. Comparative analysis of protein structure alignments. *BMC Struct Biol*, 7:1–15, 2007.

S3 Figures and Tables

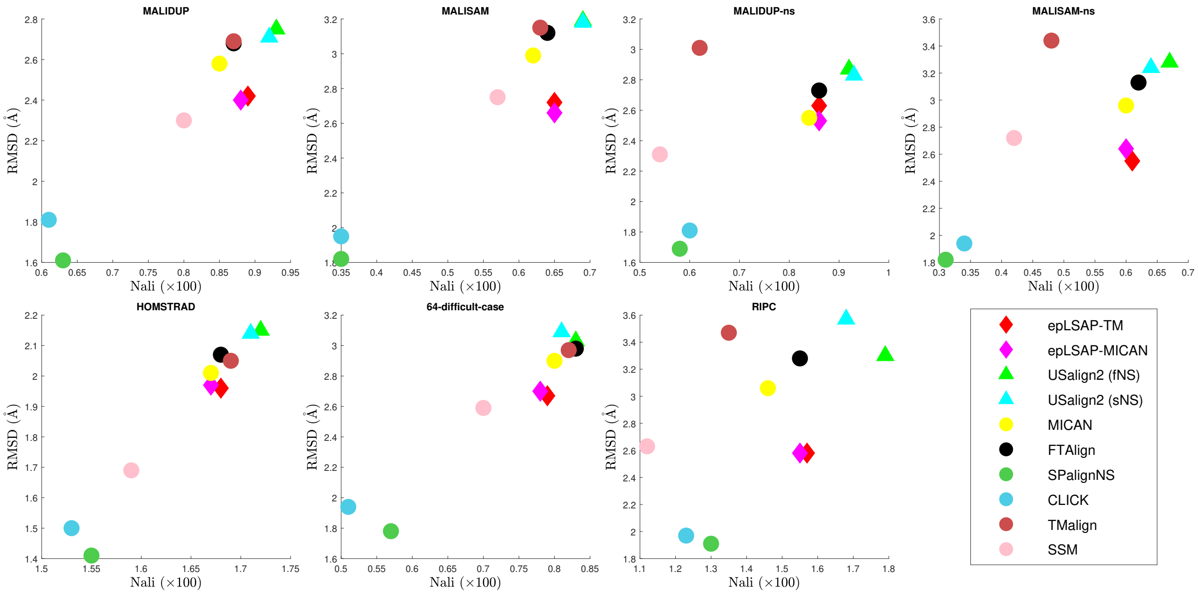

Figure S2: Plots of Nali (%)-RMSD (Å) for different methods on the seven datasets.

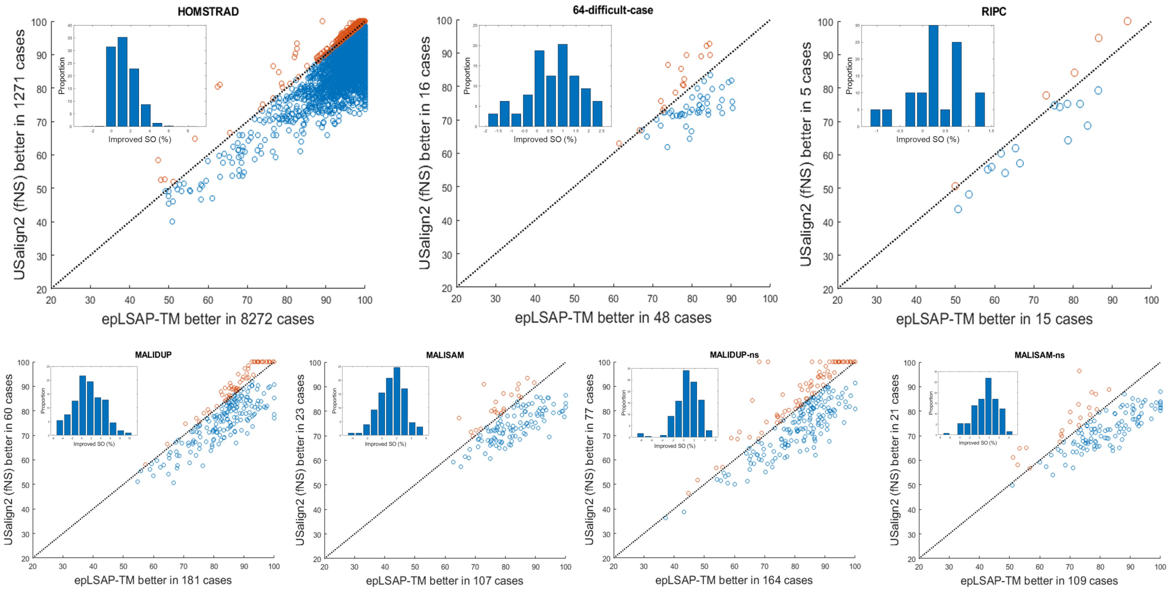

Figure S3: SO comparisons between epLSAP-TM (x-axis) and USalign2 (fNS) (y-axis) on the seven datasets. The inset figures are the distribution of improved SO of epLSAP-TM compared with the result of USalign2 (fNS).

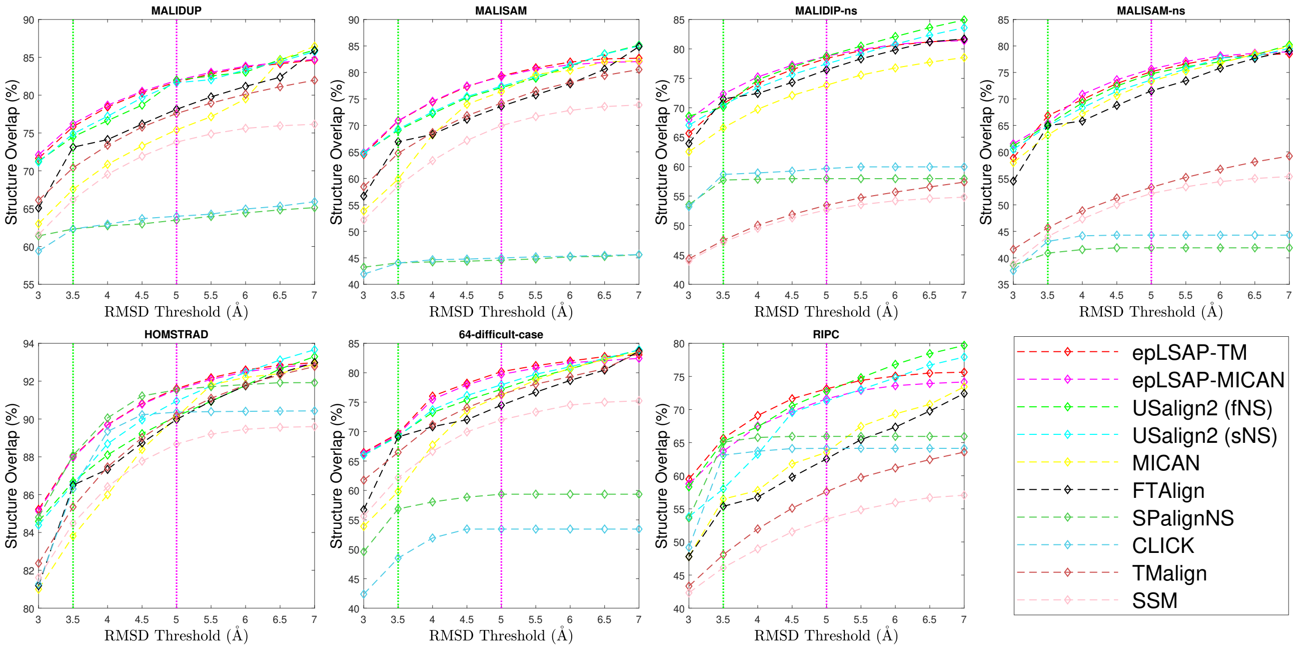

Figure S4: Study on the varying structure overlap *w.r.t.* RMSD threshold on the seven datasets. Here we take the RMSD from 3.0 to 7.0 at intervals of 0.5 and depict the curve of Eq. (9). Vertical lines at 3.5Å and 5.0Å are marked because these two thresholds are more widely-used in structure overlap quantification.

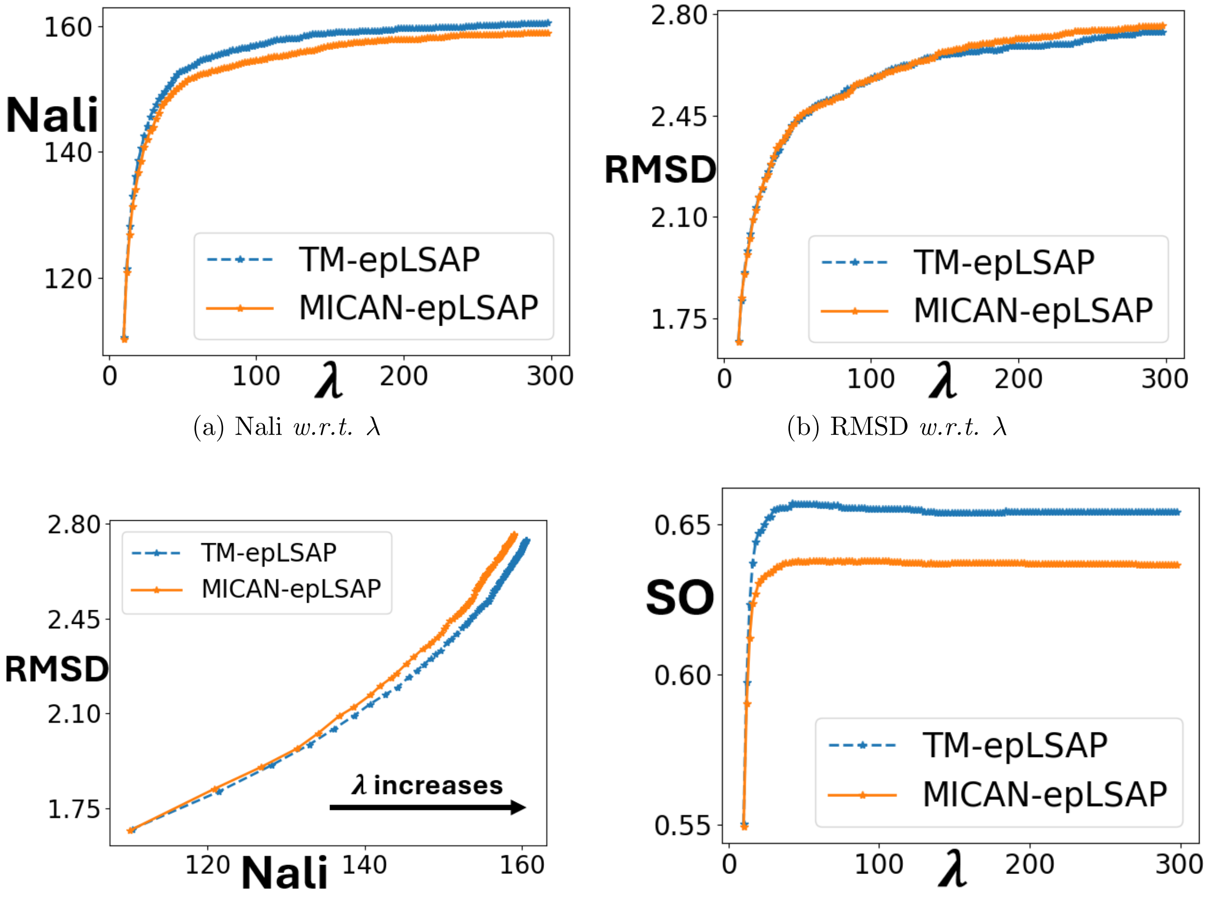

(a) Nali *w.r.t.* λ

(b) RMSD *w.r.t.* λ

(c) Nali-RMSD trade-off

(d) SO *w.r.t.* λ

Figure S5: Study on how entropy regularization controls coverage-fidelity trade-offs by taking different λ. (a) Change of Nali (coverage) *w.r.t.* λ; (b) Change of RMSD (fidelity) *w.r.t.* λ; (c) Coverage-fidelity trade-off curve *w.r.t.* varying λ; (d) Change of SO *w.r.t.* λ.

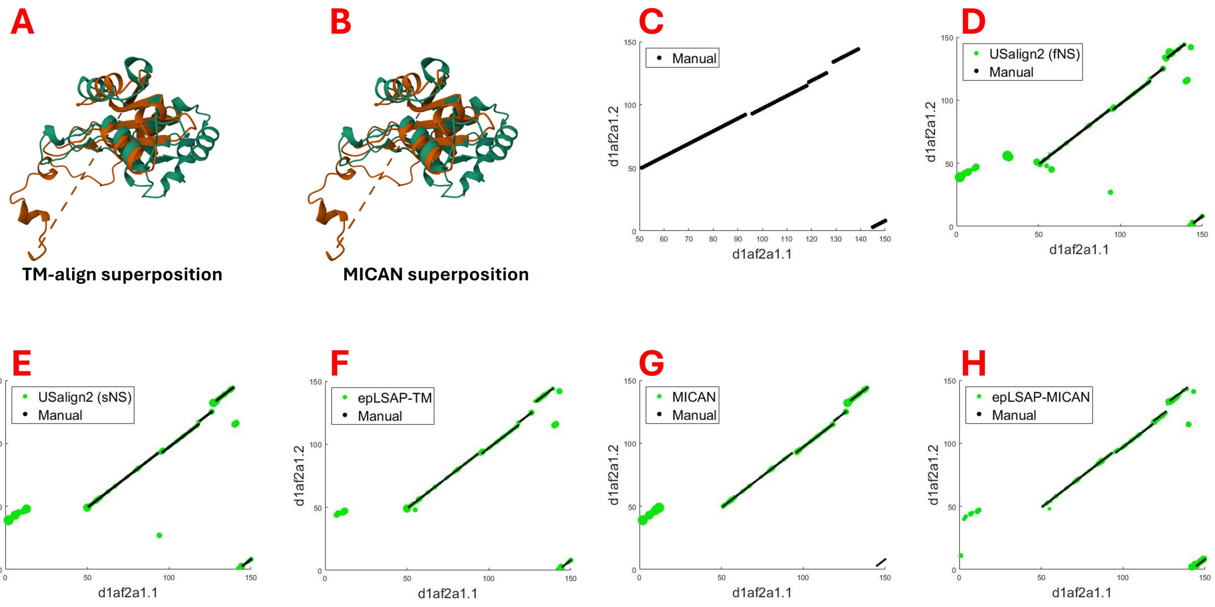

Figure S6: NS alignment example of **d1af2a1.1** (**green**)-**d1af2a1.2** (**brown**). A-B are the superposition produced by TM-align and MICAN, respectively. C is the manual ground truth produced by the multiple segment permutation technique. D-F are the alignment results of USalign2 (fNS), USalign2 (sNS) and epLSAP-TM with the same superposition of A. G-H are the alignment results of MICAN and epLSAP-MICAN with the same superposition of B. In D-H, sizes of green symbols are linearly scaled to the distance between the aligned C_α atoms.

Table S1: Comparisons between epLSAP solver and eLSAP solver

Dataset	Metric	Method			
		epLSAP-TM	eLSAP-TM	epLSAP-MICAN	eLSAP-MICAN
MALIDUP	Nali	89	97	88	96
	RMSD	2.42	2.86	2.40	2.39
	SO (%)	75.8	71.3	76.2	71.5
MALISAM	Nali	65	71	65	82
	RMSD	2.72	3.17	2.66	3.17
	SO (%)	70.8	66.8	71.0	67.3
MALIDUP-ns	Nali	86	96	86	97
	RMSD	2.63	2.93	2.53	2.89
	SO (%)	70.7	68.7	72.3	69.8
MALISAM-ns	Nali	61	68	60	69
	RMSD	2.55	3.25	2.64	3.29
	SO (%)	66.8	64.3	65.4	64.7
HOMSTRAD	Nali	168	175	167	175
	RMSD	1.96	2.16	1.97	2.18
	SO (%)	88.0	86.2	87.9	86.0
64-difficult-case	Nali	79	84	78	82
	RMSD	2.67	3.13	2.70	3.15
	SO (%)	69.7	67.5	69.5	66.4
RIPC	Nali	157	172	155	170
	RMSD	2.58	3.24	2.58	3.25
	SO (%)	65.5	63.2	63.8	62.9

Table S2: Equivalent reference residues of non-sequential alignment methods for the 20 sample pairs of RIPC dataset.

Protein pair (Total aligned number)	epLSAP -TM	epLSAP -MICAN	USalign2 -(fNS)	USalign2 -(sNS)	FTAlign	MICAN	CLICK	SPalignNS	SSM
d1qq5a__d3chy__ (3)	1	0	0	0	0	0	0	0	0
d2adma__d2hmyb_ (12)	11	12	0	12	10	12	0	10	11
d1an9a1_d1npx_1 (11)	10	10	10	7	9	11	7	8	9
d1ay9b__d1b12a_ (10)	10	10	10	10	10	10	9	10	9
d1b5ta__d1k87a2 (8)	7	8	0	0	8	8	8	0	6
d1crl___d1ede__ (3)	3	3	3	3	3	3	3	3	3
d1d5fa__d1nd7a_ (6)	4	4	1	4	4	4	3	4	4
d1dlia1_d1mv8a1 (4)	2	2	2	2	2	2	2	2	2
d1gbg___d1ovwa_ (3)	3	3	1	1	3	3	1	1	3
d1gsa_1_d2hgsa1 (5)	4	5	2	2	5	5	2	2	2
d1hava__d1kxf__ (4)	4	4	3	4	4	4	4	4	4
d1hcy_2_d1lnlb1 (4)	4	4	3	2	2	3	1	2	2
d1jj7a__d1lvga_ (8)	2	2	1	4	4	3	0	3	8
d1jwyb__d1puja_ (12)	12	10	0	11	9	4	10	9	4
d1jwyb__d1u0la2 (11)	10	11	11	11	11	11	0	0	0
d1kiaa__d1nw5a_ (12)	10	0	9	10	9	0	8	9	3
d1nkl___d1qdma1 (72)	13	21	56	64	12	26	62	52	10
d1nls___d2bqpa_ (6)	6	6	6	5	6	6	5	5	6
d1nw5a__d2adma_ (13)	2	5	1	3	6	3	0	2	0
d1qasa2_d1rsy__ (75)	74	74	72	74	68	66	69	65	63
EQR	192	194	191	229	185	184	194	192	149

Table S3: Reference-independent comparisons on **2ES9-1SXJ**

Method	Nali ↑	RMSD ↓	SO(%) ↑
epLSAP-TM	89	3.13	65.2
epLSAP-MICAN	83	2.55	70.0
USalign2 (fNS)	84	3.55	64.9
USalign2 (sNS)	77	4.37	45.4
MICAN	76	4.07	54.0
SPalignNS	65	2.10	67.1
CLICK	58	2.01	57.4
epLSAP-SP_rigid	83	2.66	68.8
